# Data Mining Climate Variability as an Indicator of U.S. Natural Gas

**DOI:** 10.3389/fdata.2019.00020

**Published:** 2019-06-20

**Authors:** Jacob Stuivenvolt-Allen, Simon S.-Y. Wang

**Affiliations:** Department of Plants, Soils and Climate, Utah Climate Center, Utah State University, Logan, UT, United States

**Keywords:** climate, natural gas, extreme climate, energy, Pacific Decadal Oscillation

## Abstract

Anomalously cold winters with extreme storms strain natural gas (NG) markets due to heightened demand for heating and electricity generation. While extended weather forecasting has become an indicator for NG management, seasonal (2–3 month) prediction could mitigate the impact of extreme winters on the NG market for consumers and industry. Interrelated climate patterns of ocean and atmospheric circulation anomalies exhibit characteristics useful for developing effective seasonal outlooks of NG storage and consumption due to their influence on the persistence and intensity of extreme winter weather in North America. This study explores the connection between the Pacific-North American climate systems and the NG market in the U.S., connecting macro-scale oceanic and atmospheric processes to regional NG storage and consumption. Western Pacific sea surface temperatures and atmospheric pressure patterns describe significant variation in seasonal NG storage and consumption. Prediction of these coupled climate processes is useful for estimating NG storage and consumption; this could facilitate economic adaptation toward extreme winter weather conditions. Understanding the implicated impact of climate variability on NG is a crucial step toward economic adaptation to climate change.

## Introduction

Natural gas (NG) in the United States is used in the industrial, electric power, residential, and transportation sectors. In 2017, the U.S. Energy Information Administration (EIA) reported that NG surpassed coal as the largest source of electricity generation and that the production and consumption of NG are expected to steadily grow. Developments in technology have led to competitive pricing locally and internationally, making NG a crucial economic resource in the national and global energy market (U. S. Energy Information Administration, 2019a).

Approximately 48% of homes in the U.S. use NG as fuel for heating, resulting in consumption spikes during the cold seasons and severe winter weather (U. S. Energy Information Administration, 2018a). As a result, seasonal cycles of NG storage observe supply increases through the warm season as NG is stockpiled for peak demand in winter. In the anomalously cold winter of January 2014, single-day consumption records were set from an arctic cold-air outbreak due to a splitting of the stratospheric polar vortex. A similar event, coined the “polar vortex,” recently occurred on January 30, 2019, again setting records for NG consumption and demand (U. S. Energy Information Administration, 2019b). Frigid temperatures and record-setting demand challenged energy and heat production, causing public utility companies in the Midwest to issue notices asking for decreased NG usage from residents and commercial entities. Subsequently, automobile manufacturers closed operations at 18 plants in the Midwest, halting operations for ~23,000 employees to ensure NG distribution to “critical infrastructures” (U. S. Energy Information Administration, 2019a).

**Figure 1:**
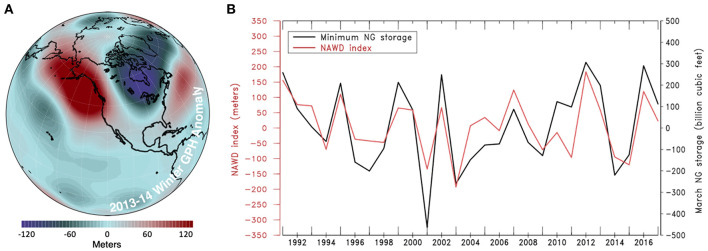
**(A)** Geopotential height (GPH) anomalies at the 250 hPa level for November–January of 2014. **(B)** Time series of annual minimum NG storage and the average November through January NAWD index (calculated by subtracting the center of the eastern trough from the center of the western ridge). The linear trend has been removed from time series data.

These record-setting consumption days are products of observed changes in atmospheric circulation. The North American Winter Dipole, a stationary “ridge-trough” pattern in the upper atmosphere, has recently been associated with severe winter weather in the U.S. (Wang et al., 2017). An example of this NAWD is illustrated by the 2013–2014 winter geopotential height anomalies at 250 hPa (Figure 1A), during the time when the western U.S. experienced severe drought while the eastern U.S. suffered from extreme cold (Singh et al., 2016; Swain et al., 2016). Structurally, the NAWD is characterized by vertically-uniform pressure anomalies of opposite sign over the Gulf of Alaska and Mid-Atlantic regions of North America. This barotropic structure is amplified in the positive phase of the dipole, suppressing cyclone-wave activity in the West (hence blocking the rainstorms) while subsequently deepening the adjacent trough in the east (Wang et al., 2014, 2015). This atmospheric pattern was also associated with the January 2018 North American Blizzard, which strained NG distribution and severely impacted consumer and market price (not shown).

This study explores the connection between the large-scale climate features of the NAWD and NG storage in the U.S. While the link between synoptic weather (like a single cold front) and NG is well understood and weather forecasting has been a tool for NG market prediction, the relationship between global-scale climate variability and NG has not been analyzed. Due to the inherently low-frequency variability of the large-scale climate systems relative to the high fluctuation of weather systems, capturing anomalous climate patterns could be conducive toward seasonal prediction of NG demand and subsequently, market price. Such information may transpire into tools for NG managers and industries to prepare for extreme winter conditions.

## Methods and Data

The following observational climate datasets were analyzed in this study: The National Center for Environmental Prediction (NCEP) / National Center for Atmospheric Research Reanalysis from 1948 to present with a 2.5° latitude and longitude resolution, the National Oceanic and Atmospheric Administration Extended Reconstructed sea surface temperature (SST) V5 with 2.0° resolution, and the Twentieth Century Reanalysis (20CR) from 1871 to 2010 with 2.0° resolution. These reanalysis and SST data were used to construct multiple linear regression models of NG storage and consumption.

For future climate assessment, large ensemble output from the Community Earth System Model (CESM) simulations under RCP8.5 (high-emission scenario) was used to analyze the NAWD and its oceanic connection through 2080. We use CESM to evaluate the projected response of these variables to a future climate scenario with continual increase in anthropogenic carbon emissions.

NG storage and consumption data is provided by the U.S. Energy Information Administration. National storage data exists from 1973 to 2018, but regional data is only available from 1990 to present. Residential and commercial consumption data is available from 1973 to present. The following study used NG storage and consumption data from the Midwest, South Central, and Eastern regions of the U.S. These areas accounted for 84% of national NG storage in 2018 and use more NG for electricity generation than the Midwest and Pacific regions. The influence of the NAWD and Western Pacific SST is most strongly related to these regions which represent the vast majority of the NG industry in the U.S.

## The North American Winter Dipole and NG Linkage

NG responded profoundly to cold-air outbreaks, as was the case during the “polar vortex” winter of 2014, driving increased demand as early as November (U. S. Energy Information Administration, 2014). Sustained cold temperatures through March drove storage levels to their lowest since 2003, while market and consumer pricing spiked. Regional consumption reached a record high for every month from November 2013 to March 2014 straining regional distribution companies and driving up price (U. S. Energy Information Administration, 2014). Many mechanisms are proposed to contribute to the weakening or splitting of the stratospheric polar vortex, but the deepened low associated with the NAWD in the Mid-Atlantic region provided the atmospheric conditions necessary for sustaining the cold-arctic air intrusion into the region (Garfinkel and Hartmann, 2008; Kim et al., 2014; Kretschmer et al., 2017). Similar conditions have been observed in the winter of 2017–2018, and the recent “polar vortex” in January of 2019. These events coincided with record NG consumption days and resulted in spikes in market and consumer price (U. S. Energy Information Administration, 2018b).

**Figure 2:**
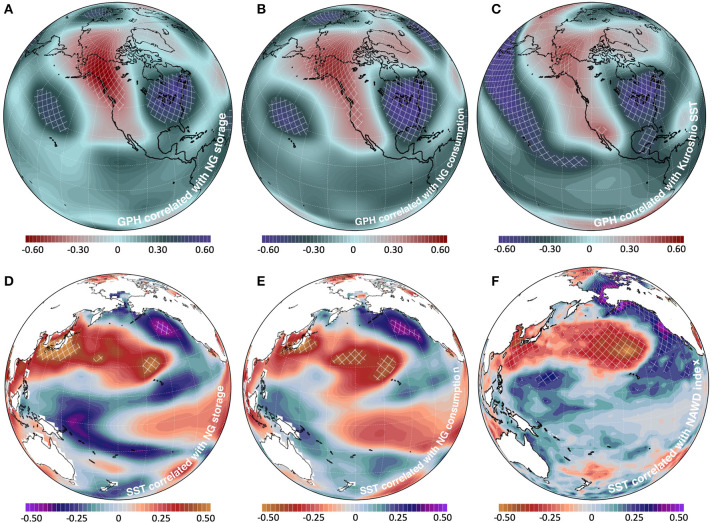
**(A)** Correlation map of GPH at 250 hPa correlated with March (minimum annual) NG storage from 1990 to 2018. **(B)** GPH at 250 hPa correlated with residential and commercial NG consumption for November through January from 1978 to 2018. **(C)** GPH at 250 hPa correlated with Kuroshio region SST for November through January from 1950 to 2014 (from twentieth Century Reanalysis). **(D)** SST correlation with NG March storage from 1990 to 2018. **(E)** SST correlated with residential and commercial consumption from 1973 to 2018. **(F)** SST correlated with the NAWD index from November through January from 1950 to 2014. The linear trend has been removed from all data.

An amplified NAWD can lead to heightened contrasts between the warm West and cold East of the U.S., creating a relationship between the NAWD and NG storage/consumption. To examine this suggested relationship between NAWD and NG, Figure 1B displays the inverse of the NAWD index, calculated by subtracting the geopotential height (GPH) of the trough center over the Great Lakes region from the ridge center in the Gulf of Alaska during the November-January season (Wang et al., 2014), with annual minimum NG storage that mostly happens in March. These time-series data are highly correlated and show that amplified conditions of the NAWD coincide with low minimum storage years for the South Central, Midwest, and Eastern NG regions (*r* = 0.766). Correlation analysis between NG storage and consumption in the same regions with the 250-hPa GPH field results in anomalous patterns over North America (Figures 2A,B) with the same structure as the amplified NAWD (Figure 1A). Correlations of NG storage and consumption for the Pacific and Mountain regions of the U.S. responded weakly to the NAWD (not shown), largely due to the mild weather induced by the western ridging. Due to the vast majority of NG consumption and storage occurring in the South Central, Midwest, and Eastern regions, the amplified NAWD apparently drives increased NG consumption and decreased supply, which has been shown to negatively impact consumer and market price in historical events (U. S. Energy Information Administration, 2014, 2018b).

## The NAWD and Climate Forcing

Western Pacific SST anomalies and Pacific climate oscillations may influence the development of the NAWD, and subsequently, winter climate in North America (Barlow et al., 2001; Taguchi et al., 2012; Wang et al., 2014; Hartmann, 2015). The Kuroshio current, the Pacific western boundary current near Japan, induces an atmospheric response that produces anticyclonic activity in the Aleutian region that can propagate into a circum-global wave train pattern (Taguchi et al., 2012). While the influence of small-scale, near-surface changes in western boundary currents and SST on basin-scale circulation is difficult to isolate, these currents affect extra-tropical climate variability (Kelly et al., 2010; Kwon et al., 2010). Wang et al. (2014) connected the NAWD amplification to abnormally warm SST in the Western North Pacific, showing that Rossby wave flux activity amplified the winter ridge in the Gulf of Alaska. Indeed, Figure 2C shows the GPH correlation pattern with SST in the Kuroshio Current region (32°-36° N, 139°-143° E) and it reveals a dipole pattern not dissimilar to those shown in Figures 2A,B, with the comparably strong low-pressure anomaly over the Great Lakes region.

The observed connections between Western North Pacific SST and the NAWD stimulate interest in viewing the coupled impact of these climate variables on winter conditions. Further correlation analysis conducted for the wintertime SST, following Figures 2A–C (changing GPH with SST), depicts a significant response in the Kuroshio Current region and the Gulf of Alaska (Figures 2D–F). Moreover, the SST patterns in North Pacific suggest a connection with the Pacific Decadal Oscillation (PDO) given the signature “horseshoe shape” of cold anomalies in the eastern North Pacific wrapping the warm anomalies extended from the Kuroshio region. The PDO is the dominant mode of monthly SST variability in the North Pacific and its formation is linked to many climate factors including teleconnections from the tropical Pacific, North Pacific atmosphere-ocean interactions, and ocean memory (Pierce, 2001; Alexander, 2010; Newman et al., 2016). Nonetheless, these significant and physically meaningful responses in SST lend support to the climate connection with NG storage and consumption, through the Western North Pacific modulation on the NAWD intensity.

## Future Projections of the NAWD

**Figure 3:**
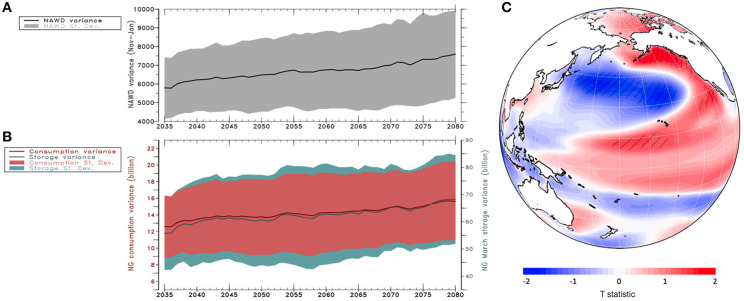
**(A)** Projected 30-year running variance of the NAWD under RCP 8.5 continued emissions scenario, calculated from CESM large ensemble (40 members). **(B)** Projected response of modeled winter (November through March) consumption and annual minimum storage under RCP 8.5. **(C)** T statistic distribution from regression of November through January NAWD index with SST for the same months of the following year. Hatched values indicate statistical significance (*p* < 0.20).

Through multiple regression of historical data, Kuroshio region SST and the NAWD describe 65% of the variance of March NG storage and 51% of the variance of winter (November–February) NG consumption. The regression equation for NG consumption and storage is shown in Equation 1 and 2 respectively. While regression analysis fails to capture some of the recent extremes in NG, these climate variables explain a significant amount of variation in a complex global market. Additionally, the correlation between Kuroshio region SST and the NAWD is a potential source of collinearity in the model, but together they represent a physically plausible link between climate and natural gas. By adopting these empirical (regression) relationships among NG, the NAWD, and Kuroshio region SST, one can assess the part of future variability in NG storage and consumption that is associated with these climate conditions. Subsequently, we analyzed the future projections from CESM output with 40 ensemble members under the RCP 8.5 continued carbon emission scenario, and the result in Figure 3A displays a universal increase in the variances of the NAWD. Figure 3B displays the increase in variance for modeled NG March storage and winter consumption driven by the NAWD and Kuroshio SST (22.2 and 24.1% increase respectively). These results suggest increased volatility that is driven by more variable climate; this project is supportive of the multi-model assessment of the NAWD by Wang et al. (2015) and the extreme water cycles in the western U.S. (Yoon et al., 2015; Swain et al., 2018). With existing management strategies, consumer and market price can be expected to respond to increased volatility in supply and demand, that is, provided that the climate conditions in terms of the NAWD and SST anomalies are properly monitored or forecasted.

(1) $$\begin{array}{l} NG\;Storage=\;877.42+\left(2329.35\right)\;*\;NDJz\\ +\left(-73540.23\right)\;*\;Kuroshio\;SST \end{array}$$

(2) $$\begin{array}{l} NG\;Consumption=3382.33+1060.76\;*\;NDJz\\ +\left(-111620.60\right)\;*\;Kuroshio\;SST \end{array}$$

**Equation 1 and 2:** Regression equations explaining 65 and 51% of the respective variance in storage and consumption. NDJz is the average value of the NAWD index from November, December, and January.

The CESM large ensemble analysis exhibits the PDO SST pattern when regressing with the NAWD in the following year (Figure 3C), suggesting that PDO in its positive phase could be a “precursor” for an amplified NAWD. This result bears similarity with the finding of Wang et al. (2014), who linked the NAWD to a type of El Niño-Southern Oscillation (ENSO) precursor with a particularly strong response in the Western North Pacific, as was depicted in Figure 2F (east of Taiwan and south of Japan). Figure 3C also is in line with previous observations (Deser et al., 2012; Hartmann, 2015) that warm SST anomalies in the tropical and North Pacific influence extreme winter weather in North America. While the CESM-simulated SST pattern associated with the NAWD a year later (Figure 3C) does not reveal a significant correlation in that particular region, persistence of the PDO could lead to ENSO in the following year(s) (Di Lorenzo et al., 2015; Matveeva et al., 2018) and the documented ability of a similar model in capturing ENSO and the PDO (Deser et al., 2012) is supportive that the air-sea interactions are reasonably simulated.

## Discussion

The incorporation of big data for the purpose of this article is related to the unstructured and seemingly unrelated nature of climate and NG in the United States. NG consumption and storage are not controlled by climate variability alone; however, the strong seasonal relationship between NG consumption and air temperature allows regional climate variability to account for a significant portion of these industries. Storage and consumption dictate the supply and demand of NG for residential and commercial use, directly impacting consumer and market price. Regional NG companies could use the projected responses of NG storage and consumption to inform long-term supply management and plan for record-breaking consumption days during extreme winter events associated with the amplified NAWD. Improved management from subseasonal climate prediction (>2 weeks) that is being actively developed (Committee on Developing a U. S. Research Agenda to Advance Subseasonal to Seasonal Forecasting, 2016), could mitigate price increases for consumers and distribution companies, compared to current methods that rely heavily on weather forecasting (<7 days).

The projected response of NG to climate variability suggests that adaptive management will be important for years to come. CESM results show increased variability in climate patterns that provide the necessary conditions for extreme weather events that coincide with antagonistic impacts on NG consumers and industry. Under the continued high-emission scenario, climate change is projected to increase energy costs through the twenty-first century by $32 billion to $87 billion (Hsiang et al., 2017). Increased demand for energy due to enhanced weather variability, increased population, and decreased water resources for hydropower generation and cooling for electricity generation could negatively impact energy producing industries (Hsiang et al., 2017).

## Data Availability

The natural gas datasets for this study can be found in the Energy Information Administration website: [Energy Information Administration] [https://www.eia.gov/naturalgas/]. Climate Reanalysis and observational is provided by the National Oceanic and Atmospheric Administration [Earth System Research Laboratory] [https://www.esrl.noaa.gov/psd/data/gridded/]. CESM model output data is available from UCAR/NCAR [Community Earth System Models] [http://www.cesm.ucar.edu/].

## Author Contributions

SW is responsible for the original analysis connecting the North American Winter Dipole to natural gas storage. SW advised and oversaw the rest of the analysis which was done in tandem with JS-A. The manuscript and figures were prepared by JS-A and reviewed by SW.

### Conflict of Interest Statement

The authors declare that the research was conducted in the absence of any commercial or financial relationships that could be construed as a potential conflict of interest.
